# The Treatment of Syphilis

**Published:** 1911-08

**Authors:** W. W. Quinton

**Affiliations:** Buffalo, N. Y.; 232 Elmwood Avenue


### The Treatment of Syphilis

By W. W. QUINTON, M.D.
Buffalo, N. Y.

THE present status of the treatment of Syphilis is of the greatest interest to the general practitioner as well as to the specialist.

The sale of Erlich's Salvarsan is now unrestricted and if this valuable remedy is indiscriminately and carelessly used we may expect deplorable results.

It has been heralded far and near as a sure cure for Syphilis and demands for its administration will be made by many unfortuntes suffering with the disease, regardless whether or not the remedy is indicated.

Even with the most carefully selected cases and the most perfect technic bad results have occurred and these should warn us to regard the use of this drug in a most conservative spirit, relying mainly on the older methods of treatment and using Salvarsan only in cases where it is plainly indicated, meanwhile patiently awaiting the time when careful experimentation shall have taught us the indications and the contraindications of this extremely valuable remedy.

The older methods, when properly applied, have given satisfactory results and the cures resulting while slow have been sure.

What treatment shall we use until the status of '606' has been determined?

Both at home and abroad many authorities look upon the salicylate of mercury as the best drug for ordinary use. It is administered with sterilised liquid paraffin in 10 per cent. strength. The dose is 1 cc. injected into the buttock. This injection is given weekly for ten weeks which constitutes a main cure.

Some patients do not support the salicylate and suffer with severe headache, profuse sweating, ringing in the ears, and the like. In these cases the thymol acetate can be substituted, using it in the same strength.

How long will it take to cure a patient by this method, and when can he be assured that it will be safe for him to marry?

In order to bring about a positive and lasting cure it is necessary that the patient should be under observation for three years. Three main cures are given in the first year, two in the second year and two in the third year. If the patient takes all of the main cures and remains at the end of the third year without symptoms and with a negative Wassermann, he is cured.

The succinimide of mercury may be used instead of the salicylate. It is injected in 1 per cent. solution in distilled water with the addition of a small amount of cocaine to prevent pain. 1 cc. of the solution is injected daily; thirty injections constitute a main cure. This solution must be renewed every eight days.

The difference between the two preparations can be summed up in a few words. Soluble salts of mercury as the succinimide, cause a very quick effect; a large amount of mercury being absorbed in a short time. The insoluble salts, as for example the salicylate, give a slow continuous action during a protracted period.

In cases of brain syphilis, where a very energetic effect is desired, calomel can be employed in 5 per cent. strength in sterilised liquid paraffin. This is one of the most powerful preparations of mercury.

The perchloride of mercury is very speedy in its effects. The objections to its use are that it is very painful, may cause abscess and destroys the needle.

A 2 per cent. solution is administered every other day or a 5 per cent. solution every five days. The disadvantages attending its use are very evident but it should be remembered that in syphilitic apoplexy, gumma of the brain or in arteritis syphilitica a 5 per cent. solution used judiciously may save the patient.

When given by intravenous injection it is not painful and the danger of abscess formation is obviated. A Platino-iridium needle of Merck or Beyer make should be provided.

Énésol which is a combination of arsenic and the salicylate of mercury is a valuable preparation particularly in anemic cases.

It might be well to mention the fact that such a thing as mercurial saturation exists and when this occurs further dosage with the drug will have no effect. In these cases the mercury must be stopped for a time.

If symptoms persist between the cures, other forms of mercury can be given combined with the iodides.

The iodides are always given after each main cure with mercury, as they seem to have the effect of fixing the mercury in the system.

The iodide of sodium is preferred as it is better borne by the stomach. It contains approximately the same percentage of iodine as the iodide of potassium.

The special indications for the use of the iodides are:

1. Pain.

2. Gummatous destruction of the nose or throat, in which case we give the iodides first.

3. Gumma on the leg. The iodides are employed internally and mercurial plaster locally.

4. In cases of mercurial idiosyncrasy.

In the course of treatment with the iodides the patient must be watched carefully for symptoms of poisoning, such as eruption, lacrimation, scratching in the throat, and the like. Eighty per cent. of the patients will sooner or later develop some slight symptoms of iodism but if severe, the iodides must be stopped, for persistence in their administration has caused death.

In severe headache and boneache nothing will prove more beneficial than an injection of a mixture of iodoform and sterilised liquid paraffin 1-10. One, two or three injections of 1 cc. each are given intramuscularly.

Mucus patches are touched with nitrate of silver, or the bichloride of mercury 1-100. When the whole mouth is affected a spray containing 1 part of iodoform in fifty parts each of ether and alcohol will be found beneficial.

For leucoplakia, painting three or four times a day with balsam of peru is strongly recommended.

In all local skin affections mercurial plaster may be employed.

The treatment of the initial lesion calls for a few comments. There is great difference of opinion on this point but it is believed that primary excision of the chancre may prove helpful.

In syphilitic alopecia daily massage with an ointment of ammoniated mercury five parts to fifty will be found of service.

In ozena, peroxide of hydrogen used as a spray will do much towards relieving the condition.

The palmar and plantar lesions can be treated with bichloride of mercury in collodion 1-100.

These and many other methods of treatment have met with success in dealing with the disease but in face of all that is claimed for the new drug, the Erlich-Hata "606" or Salvarsan, they appear slow and ineffectual by comparison.

It must be remembered however, that cases which have received careful and intelligent treatment by the older methods are cured and remain so.

One of the greatest dangers to be apprehended from the new treatment seems to me to lie in the fact that so many patients will expect a "one injection" cure. This injection may clear up all the local lesions and the patients will go their several ways confident that a cure has been obtained, only to report in the course of time with the tertiary manifestations.

The time may come when we shall use "606" to the exclusion of all other methods of treatment but that day has not arrived.

The drug may now be used in certain selected cases in which the presence of the spirocheta has been definitely determined. These cases should be kept under observation for long periods of time and frequently tested with the Wassermann until it is known that a cure has been effected.

Certain phases of the disease will indicate the administration of Salvarsan at the present time, viz:

1. Refractory cases which do not respond to mercury.
2. Relapse following mercurial treatment.
3. Cases with mercurial idiosyncrasy.
4. Those which are continually relapsing.
5. In malignant syphilis.
6. Cases complicated by tuberculosis.
7. For the relief of pain in some cases of early tabes.
8. Hereditary syphilis in infants.
9. In the primary attack, given at the appearance of the chancre, and followed by prolonged and intermittent treatment with mercury accompanied by local treatment of the initial lesion.

The drug, however, must be used with great care. The patients must be selected from those who are otherwise strong and healthy. Cachexia or a weakened constitution will always contraindicate its use. The eye should be normal; temperature and body weight should be determined and the urine examined.

Salvarsan must not be used in old age or in affections other than syphilis of the kidney, heart, liver, spleen, lung, and blood-vessels, or in grave syphilitic affections of the brain, as recent hemiplegia or acute or subacute meningitis.

It should not be administered in paresis or advanced tabes or to those with advanced degenerative changes of the brain from any cause. Acute febrile disturbances and chronic alcoholism will also act as contraindications.

It would seem that the ideal treatment of syphilis in the future will result from a combined treatment of selected cases with Salvarsan and mercury. The Salvarsan to be given as soon as the presence of the spirocheta has been determined, to clear up quickly the primary manifestations and render the possibility of contagion less likely. Then following this a vigorous intermittent treatment of the disease by mercurial injections with the frequent use of the Wassermann reaction until the patient is cured.

This will insure a shorter period of treatment, the greater safety of those with whom the patient may come in contact and an eventual and lasting cure of the disease.

232 Elmwood Avenue.

